# Two Strains of *Lentinula edodes* Differ in Their Transcriptional and Metabolic Patterns and Respond Differently to Thermostress

**DOI:** 10.3390/jof9020179

**Published:** 2023-01-29

**Authors:** Yuan Guo, Qi Gao, Yangyang Fan, Shuang Song, Dong Yan, Jing Zhao, Yulin Chen, Yu Liu, Shouxian Wang

**Affiliations:** 1 Beijing Engineering Research Center for Edible Mushroom, Institute of Plant Protection, Beijing Academy of Agriculture and Forestry Sciences, Beijing 100097, China; 2 College of Horticulture and Plant Protection, Inner Mongolia Agricultural University, Hohhot 010018, China; 3 College of Agriculture and Food Engineering, Baise University, Baise 533000, China

**Keywords:** *Lentinula edodes*, temperature type, heat stress, multi-omics, transcriptome, metabolome

## Abstract

Temperature type is one of the key traits determining the cultivation regime of *Lentinula edodes*. However, the molecular and metabolic basis underling temperature type remain unclear. Here, we investigated the phenotypic, transcriptomic, and metabolic features of *L. edodes* with different temperature types under both control (25 °C) and high (37 °C) temperature conditions. We found that under the control condition, the high- and low-temperature types of *L. edodes* harbored distinct transcriptional and metabolic profiles. The high-temperature (H-)-type strain had a higher expression level of genes involved in the toxin processes and carbohydrate binding, while the low-temperature (L-)-type strain had a high expression level of oxidoreductase activity. Heat stress significantly inhibited the growth of both H- and L-type strains, while the latter had a higher growth inhibition rate. Upon exposure to heat, the H-type strain significantly up-regulated genes associated with the components of the cellular membrane, whereas the L-type strain markedly up-regulated genes involved in the extracellular region and carbohydrate binding. Metabolome data showed that thermostress altered purine and pyrimidine metabolism in the H-type strain, whereas it altered cysteine, methionine, and glycerophospholipid metabolism in the L-type strain. Transcriptome and metabolome integrative analysis was able to identify three independent thermotolerance-related gene–metabolite regulatory networks. Our results deepen the current understanding of the molecular and metabolic basis underlying temperature type and suggest, for the first time, that thermotolerance mechanisms can be temperature-type-dependent for *L. edodes*.

## 1. Introduction

*Lentinula edodes* (Berk.) Pegler is a white-rot fungus broadly distributed in the subtropical to temperate regions of the of North and South America, Asia, and Australia [1], particularly in China and Japan [2,3]. It is known as “shiitake” in Japan and “Xianggu” in China. It is the second most cultivated and the most popular edible fungus in the world [4,5]. Except for its important role as food, *L. edodes* possesses huge potential for therapeutic applications due to its abundant bioactive components including polysaccharides, sulfur-compounds, phenolics, flavonoids, etc. [6,7,8].

It is estimated that there are approximately 500 *L. edodes* cultivars present in China [9]. Based on the optimal temperature range for fruiting body formation, *L. edodes* can be classified into different temperature types including the high-temperature type (H-type, fruiting at 15–25 °C), medium-temperature type (M-type, fruiting at 10–20 °C), low-temperature type (L-type, fruiting at 5–15 °C) and broad-temperature type (B-type, fruiting at 5 to 25 °C) [10,11]. Temperature type is the key trait in determining the regional cultivation regimes of *L. edodes* [10,11]. Temperature type is also among one of the most important traits for breeding purposes. The temperature type of *L. edodes* can be characterized by different strain-typing approaches including amplified fragment length polymorphism (AFLP) markers and inter-simple sequence repeat markers (ISSR), suggesting that strains with the same temperature type may share the same gene pool [10,12]. However, the genetic basis underlying temperature type remains largely unknown [11,13]. More recently, it was reported that temperature is the key environmental factor involved in the genetic divergence and phenotypic differentiation of *L. edodes* from the aspect of population genomics [14]. Therefore, it is of great value to uncover the molecular basis behind temperature type.

Heat stress is a major abiotic constraint in mycelial growth and fruiting body productivity for macrofungi [15,16]. Direct injuries that result from high temperatures include cell wall damage [17], increased membrane fluidity and permeability [18,19], nuclear condensation, mitochondrial dysfunction, and DNA fragmentation [20,21]. Thermostress also causes cell metabolism disorder as microorganisms typically respond to stress through the reprogramming of metabolic profiles [16]. In macrofungi, thermostress has been found to alter metabolic pathways including the tricarboxylic acid cycle, glucose metabolism, sphingolipid metabolism, and some amino acid metabolism [21,22,23]. Indirect impacts include increased susceptivity to fungal pathogens [24]. In addition, heat stress could also inhibit the growth and development of *L. edodes* by reducing carbon availability [25,26]. The molecular mechanisms by which macrofungi address thermostress comprise activation of the antioxidant defense system, the synthesis of trehalose and auxins, the expression of heat shock proteins (HSPs), etc. [16]. In *Pleurotus eryngii*, Kong et al. found that nitric oxide may function as a signaling molecule to mitigate HS-induced oxidative damage [27]. Upon exposure to heat, heat response factors (HSFs) can boost the transcription and accumulation of heat-stimulated gene products by interacting with heat stress-related genes [28]. In *L. edodes*, it was reported that HSPs could enhance thermotolerance by regulating IAA biosynthesis [15,25,29,30,31]. To date, however, the differences in gene expression and metabolic adaptation for different temperature types of *L. edodes* in response to high temperature stress have not yet been studied.

In this study, we compare the phenotypic, gene expression, and metabolic profiles of two widely cultivated H- and L-type *L. edodes* strains in China under normal (25 °C) and high (37 °C) temperature conditions using RNA-Seq and liquid chromatography–mass spectrometry (LC-MS)-based metabolomics analysis. We found that different temperature types of *L. edodes* differ in their transcriptional and metabolic profiles. Upon exposure to heat, H- and L-type strains responded differently at the level of the transcriptome and metabolome. These results deepen our understanding of the molecular and metabolic basis underlying the temperature type of *L. edodes*, and suggest that the thermotolerance mechanisms might be temperature-type-dependent. The data also indicate that the temperature types might be associated with the thermotolerance of mycelium.

## 2. Materials and Methods

### 2.1. Fungal Strains and Cultivation

The *L. edodes* strains JZB2102217 (H-type) and JZB2102031 (L-type) used in this study were supplied by the Beijing Germplasm Resource Bank for Edible Fungi. The two selected strains are among the typical H- and L-type strains widely cultivated in northern and southern China. Mycelia were punched out using a cork borer (1 cm diameter), and then, were inoculated in Petri dishes (10 cm diameter) containing 35 mL of potato dextrose agar (PDA) medium as described previously [32]. Prior to inoculation, a sterilized cellophane membrane was placed on the surface of the PDA medium for easier collection of the mycelium samples. The two strains were cultivated in a growth incubator at 25 °C and in permanent darkness. After 5 days of growth at 25 °C, the fungal cultures were divided into the control and treatment groups, where the latter were subjected to heat exposure at 37 °C for 24 h.

### 2.2. RNA Isolation, cDNA Library Construction, and Illumina Hiseq X Ten/Nova seq 6000 Sequencing

Total RNA was extracted in triplicate from the mycelia of *L. edodes* using TRIzol^®^ Reagent according to the manufacturer’s instructions (Invitrogen, Carlsbad, CA, USA). Genomic DNA was removed using DNase I (Takara, Kyoto, Japan). RNA quality was evaluated using the Agilent 2100 BioAnalyzer (Agilent Technologies, Palo Alto, CA, USA) and quantity was determined using ND-2000 (NanoDrop Technologies, Wilmington, DE, USA). High-quality RNA samples (OD260/280 = 1.8–2.2, OD260/230 ≥ 2, RIN ≥ 6.5, 28S:18S ≥ 1.0, > 1 µg) were used to construct a sequencing library.

RNA-seq transcriptome libraries were prepared following the instructions of the TruSeq^TM^ RNA sample preparation Kit from Illumina (San Diego, CA, USA) using 1 μg of total RNA. Briefly, mRNA, which was enriched by poly A tail selection and chemically fragmented, was used for first-strand cDNA synthesis, followed by second-strand cDNA synthesis using a SuperScript double-stranded cDNA synthesis kit (Invitrogen, Carlsbad, CA, USA) with random hexamer primers (Illumina, San Diego, CA, USA). Then, the synthesized cDNA was subjected to end-repair, phosphorylation, and ‘A’ base addition according to Illumina’s library construction protocol. The libraries were then size-selected for cDNA target fragments of 300 bp on 2% low-range ultra-agarose followed by PCR, amplified using Phusion DNA polymerase (NEB, Ipswich, MA, USA) for 15 PCR cycles. After quantification by TBS380, the paired-end RNA-seq sequencing library was sequenced using the Illumina HiSeq X Ten/NovaSeq 6000 sequencer (2 × 150 bp read length) (Illumina, San Diego, CA, USA).

### 2.3. Read Mapping

The raw paired-end reads were trimmed and quality-controlled using SeqPrep (https://github.com/jstjohn/SeqPrep (accessed on 25 March 2022)) and Sickle (https://github.com/najoshi/sickle (accessed on 25 March 2022)) with default parameters. Then, clean reads were separately aligned to the reference genome (GCA_015476405.1) in orientation mode using HISAT2 (http://ccb.jhu.edu/software/hisat2/index.shtml (accessed on 25 March 2022)) software [33]. The mapped reads of each sample were assembled using StringTie (https://ccb.jhu.edu/software/stringtie/index.shtml?t=example (accessed on 25 March 2022)) in a reference-based approach [34].

### 2.4. Differential Expression Analysis and Functional Enrichment

For gene expression analyses, fragments per kilobase per million reads (FPKM) values were calculated using RSEM software (Version 1.3.3, http://deweylab.biostat.wisc.edu/rsem/ (accessed on 25 March 2022)) [35]. Differentially expressed genes (DEGs) were identified using DESeq2 [36], in accordance with the following general criteria:|log2FC| > 1 and padjust ≤ 0.05. padjust was calculated using the BH (FDR correction with Benjamini–Hochberg) methods [37]. Gene ontology (GO) and Kyoto Encyclopedia of Genes and Genomes (KEGG) pathway functional enrichment analyses were performed via Goatools (Version 0.6.5, https://github.com/tanghaibao/Goatools (accessed on 25 March 2022)) and KOBAS (Version 2.1.1, http://kobas.cbi.pku.edu.cn/home.do (accessed on 25 March 2022)) [38].

### 2.5. Metabolite Extraction

The mycelium (50 mg) was homogenized in 2 mL of polypropylene in a tube under cryogenic conditions at −10 °C (50 Hz) for 6 min using a high-throughput tissue crusher Wonbio-96c (Shanghai Wanbo Biotechnology co., LTD, Shanghai, China). For extraction, 400 µL of methanol: water (4:1, *v*/*v*) extraction solvent mixture was added to the tube containing mycelium samples and 10 µL of internal standard (2-Chloro-L-Phenylalanine, HPLC hyper grade, Merck, Darmstadt, Germany, 0.02 mg/mL). Samples were sonicated at 40 kHz in an ultrasonic bath for 30 min at 5 °C, and then, kept at −20 °C for 30 min to precipitate proteins. The solution was centrifuged at 13,000× *g* at 4 °C and the supernatant was recovered for further metabolomics analysis.

### 2.6. Non-Target Metabolomics

Metabolites were chromatographically separated using an ultra-high-performance liquid chromatography (UHPLC) system (Thermo Electron Corporation, San Jose, CA, USA). The UHPLC system was equipped with an ACQUITY BEH C18 column (100 mm × 2.1 mm i.d., 1.7 µm; Waters, Milford, MA, USA). The mobile phases consisted of 0.1% formic acid in water (solvent A) and 0.1% formic acid in acetonitrile: isopropanol (1:1, *v*/*v*) (solvent B). The gradient elution program was set as follows to equilibrate the systems: from 0 to 3 min, 95% (A): 5% (B) to 80% (A): 20% (B); from 3 to 9 min, 80% (A): 20% (B) to 5% (A): 95% (B); from 9 to 13 min, 5% (A): 95% (B) to 5% (A): 95% (B); from 13 to 13.1 min, 5% (A): 95% (B) to 95% (A): 5% (B); and from 13.1 to 16 min, 95% (A): 5% (B) to 95% (A): 5% (B). The sample injection volume was 2 µL, the flow rate was 0.4 mL min^−1^, and the column temperature was kept at 40 °C throughout the chromatographic separation.

The mass spectra of the compounds were obtained using a Thermo UHPLC-Q Exactive Mass Spectrometer (MS/MS) equipped with an electrospray ionization (ESI) source (Thermo Electron Corporation, San Jose, CA, USA). The optimized parameters were set as follows: aus gas heater temperature, 400 °C; sheath gas flow rate, 40 psi; aus gas flow rate, 30 psi; ion-spray voltage floating (ISVF), −2800 V in negative mode and 3500 V in positive mode, respectively; and normalized collision energy, 20–40–60 V rolling for MS/MS. Data acquisition was performed in Data Dependent Acquisition (DDA) mode. The detection was carried out over a mass range of *m*/*z* 70 to 1050. The samples were analyzed in both positive (+) and negative (−) ESI modes.

The obtained raw data were analyzed using Progenesis QI 2.3 software (Nonlinear Dynamics, Waters, USA) to perform the peak detection, alignments, integration, isotope filtering, and peak-grouping based on peak-area correlation [39]. The preprocessed data table contains the retention time (RT), mass-to-charge ratio (*m*/*z*) value, and peak intensity. The metabolic features detected to have values of at least 80% in any set of samples were retained. After filtering, the minimum metabolite values were imputed for specific samples in which the metabolite levels fell below the lower limit of quantitation, and each metabolic feature was normalized by sum. The internal standard was used for data QC (reproducibility). Metabolic features that had a relative standard deviation (RSD) of QC > 30% were discarded. Data were logarithmically (log10) transformed prior to conducting multivariate analysis [40]. Compounds were identified via comparison of accurate mass, MS/MS fragment spectra, and isotope ratio difference against the biochemical databases the Human Metabolome Database (HMDB) (http://www.hmdb.ca/ (accessed on 10 March 2022)) and the Metlin database (https://metlin.scripps.edu/ (accessed on 10 March 2022)). The mass tolerance between the measured *m*/*z* values and the exact mass of the components of interest was ±10 ppm. Mass features without MS/MS spectra were tentatively annotated at the MS1 level using a 5.0 mDa tolerance. The metabolite abundances were quantified according to their peak areas.

### 2.7. Differential Metabolite Analysis

Orthogonal partial least squares discriminant analysis (OPLS-DA) was used to determine the differential expressed metabolites between pairwise groups. Differentially expressed metabolites (VIP ≥ 1, *p* ≤ 0.05) between groups were mapped into biochemical pathways through metabolic enrichment and pathway analysis based on a database search (KEGG, http://www.genome.jp/kegg/ (accessed on 10 March 2022)). scipy.stats (Python packages) (https://docs.scipy.org/doc/scipy/ (accessed on 10 March 2022)) was used to identify statistically significantly enriched pathway using Fisher’s exact test.

### 2.8. Reactive Oxygen Species Detection and Growth Rate Measurement

Reactive oxygen species (ROS) production was measured according to the method described previously in [41,42]. For fluorescence detection, sterile glass coverslips were obliquely inserted into the Petri dishes just after the inoculation of fungal blocks, which we allowed the fungal mycelium grow on later. When the hyphae grew on the coverslips, the coverslips with hyphae were then incubated in 10 μmol/L of 2′,7′-dichlorodihydrofluorescein diacetate (DCHF-DA, Solarbio, Beijing, China) phosphate-buffered saline (PBS) solution for 25 min under dark conditions for ROS visualization. After staining, ROS production was visualized using a fluorescence microscope (Olympus, IX71, Tokyo, Japan). The mean fluorescence intensity, i.e., the mean pixel intensity over all the pixels in the region of fluorescence, was calculated using the ImageJ program (Version 1.53t, https://imagej.nih.gov/ij/index.html (accessed on 27 December 2022)) [43]. The inhibition rate was calculated as follows: $\mathrm{growth}\;\mathrm{inhibition}\;\mathrm{rate}=\frac{G1-G2}{G2}\times 100\%$, where G1 and G2 denote the growth rate of the fungal colony after and before heat treatment. Growth rate was calculated according to the diameter change during the period of growth before and after heat treatment (mm/d).

### 2.9. Statistical Analysis

Orthogonal partial least squares regression discriminant analysis (OPLS-DA) was performed using SIMCA 14.1 (Umetrics, Umeå, Sweden). The robustness of the OPLS-DA models was assessed via seven-fold cross-validation [44]. The reliability of the predictive models was assessed via the analysis of variance testing of cross-validated predictive residuals (CV-ANOVA), R2 and Q2, which provide information on interpretability and predictability, respectively. The *p* value was estimated using a paired Student’s t-test via single-dimensional statistical analysis. To test the overall associations between DEGs and DEMs, we performed Procrustes analysis in R using the ‘vegan’ package (version 4.2.0) [45,46]. The data were logarithmically (log10) transformed, centered, and Pareto-scaled prior to multivariate analyses [40]. For the integration and visualization of transcriptome × metabolome associations, the sparse partial least squares (sPLS) regression method [47] was implemented using the R package mixOmics [48].

## 3. Results

### 3.1. H- and L-Type L. edodes Strains Differ in Their Transcriptional and Metabolic Profiles

**Figure 1:**
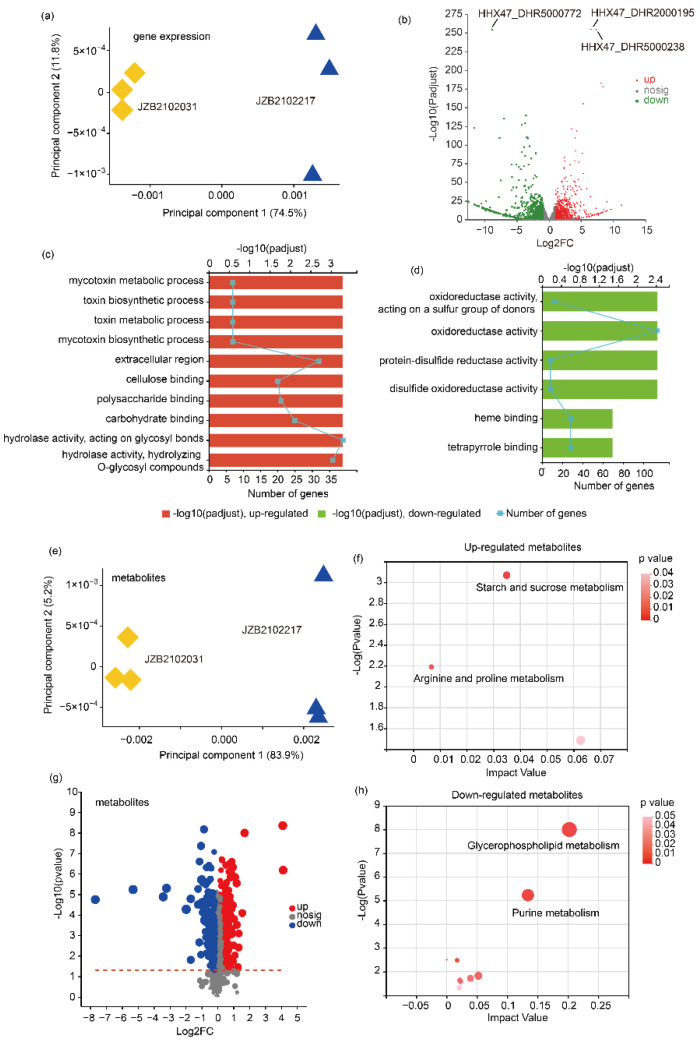
The transcriptional and metabolic profiling of H-type *L. edodes* strain JZB2102217 and L-type strain JZB2102031. (**a**) Principal component analysis (PCA) of transcriptome data of both strains; (**b**) volcano plot showing the up- and down-regulated genes; (**c**,**d**) the top 10 KEGG-enriched items of up- and down-regulated genes (padjust < 0.05), respectively; (**e**) PCA of the metabolome data of both strains; (**f**,**h**) the KEGG topology analysis of the up- and down-regulated metabolites, respectively; (**g**) volcano plot showing the up- and down-regulated metabolites (FDR < 0.05, VIP from OPLS-DA ≥ 1, |log2FC| > 1).

Transcriptomics and metabolomics were used to unearth the differences between *L. edodes* strains of different temperature type. A total of 7959 expressed genes (Table S1, RPKM ≥ 10) and 1594 metabolites (Table S2) were identified from the examined samples. PCA was used to visualize and evaluate the overall differences in gene expression and metabolics between H- and L-type *L. edodes* strains. For transcriptomics data, the first two PCs explained 86.3% of the variation; the first PC, which explained 74.5% of the total variation, was able to separate H- and L-type *L. edodes* strains (Figure 1a). A volcano plot shows the differentially expressed genes between H- and L-type strains (Figure 1b). Compared to the L-type strain, the up-regulated genes in the H-type strain were enriched mainly in toxin processes and carbohydrate binding, whereas the L-type strain had a high expression level mainly of oxidoreductase activity (Figure 1c,d).

The PCA analysis based on the detected metabolites was also able to discriminate H- and L-type *L. edodes* strains (Figure 1e). Compared to the L-type strain JZB2102031, the H-type strain JZB2102217 accumulated a significantly higher abundance of compounds enriched in starch and sucrose metabolism, and arginine and proline metabolism, while accumulating a lower abundance of compounds enriched in glycerophospholipid and purine metabolism (Figure 1f,h) based on the KEGG topology analysis (padjust < 0.05, VIP ≥ 1, |log2FC| > 1). The KEGG enrichment analysis of the up- and down-regulated genes is shown in Figure S1.

### 3.2. Heat Stress Inhibited the Growth and Induced the Production of ROS in Both Temperature Types of L. edodes

**Figure 2:**
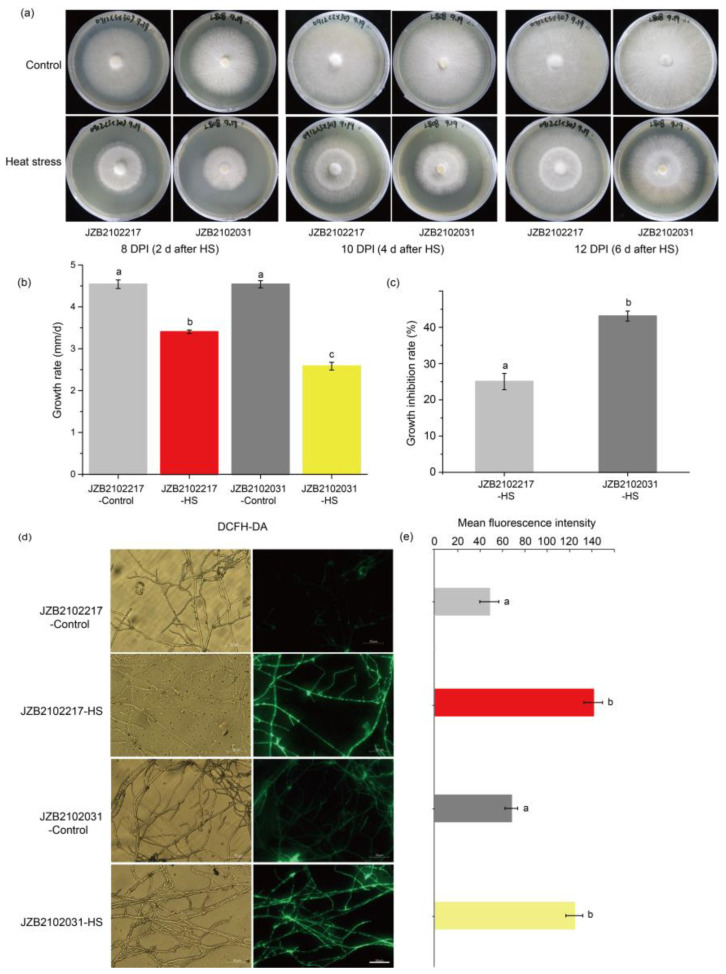
The phenotypic changes in *L. edodes* H-type strain JZB2102217 and L-type strain JZB2102031 under control (25 °C) and heat stress (HS, 37 °C) conditions. HS was conducted on the 5th day for 24 h. The fungal hyphae of two strains were able to recover growth 2 days after HS. (**a**) The morphology of cultures of H-type strain and L-type strain 2, 4, and 6 d after HS, i.e., 8, 10, and 12 days post-inoculation (DPI); (**b**,**c**) the growth rate and growth inhibition rate of two strains in response to heat stress, respectively; (**d**,**e**) the ROS production staining (Bar = 50 μm) and mean fluorescence intensity, respectively. Different letters indicate significant differences between groups (ANOVA, *p* < 0.05). Data are represented as mean ± se (*n* = 4).

Figure 2a shows the morphology of the H-type strain JZB2102217 and the L-type strain JZB2102031 under control (25 °C) and HS (37 °C) conditions. We can observe that both strains could recover growth 2 d after heat treatment. According to the growth rate, heat stress significantly suppressed the growth of both the H- and L-type strains (Figure 2a,b), while the latter grew significantly slower as a result of heat stress. After the recovery of growth, the L-type strain had a higher growth inhibition rate than the H-type strain (Figure 2c). Under optimal growth conditions, the H- and the L-type strains had comparable levels of ROS production, which was significantly induced by heat stress (Figure 2d,e). 

### 3.3. H- and L-type L. edodes Strains Varied in Their Transcriptional Profiles in Response to Heat Stress

**Figure 3:**
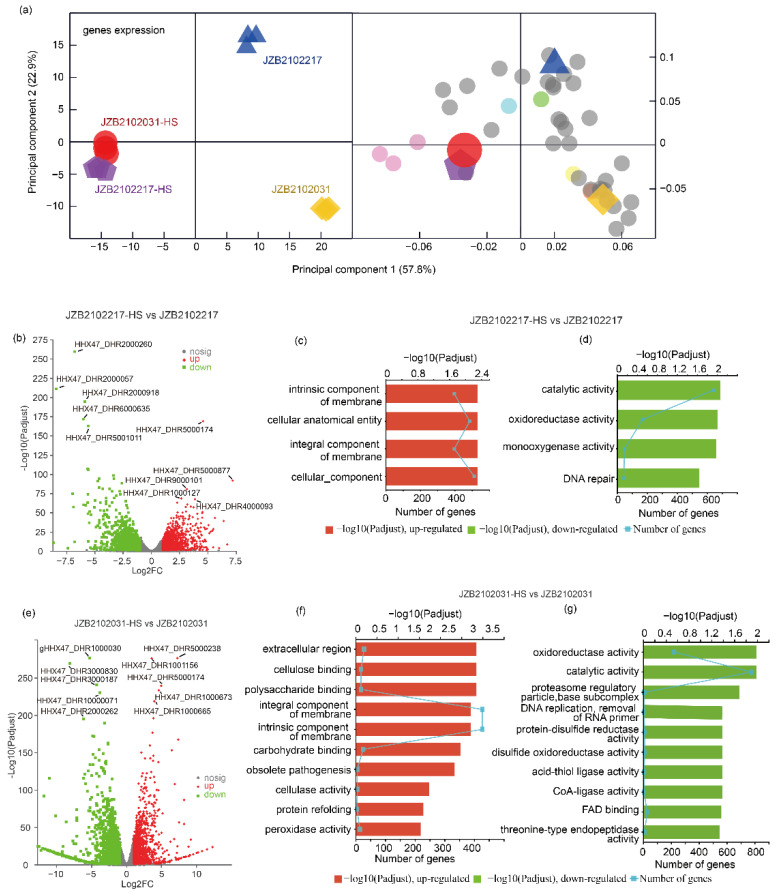
Gene expression analysis of H- and L-type *L. edodes* strains JZB2102217 and JZB2102031 in response to heat stress. (**a**) OPLS-DA analysis of transcriptome data of all samples (R2Xcum = 0.891, R2Ycum = 0.994, Q2cum = 0.931, CV-ANOVA, *p* = 9.835 × 10^−7^). OPLSDA loading showing the genes whose VIP > 4; color indicates the KEGG pathways (light bule—endocytosis; light orange—methane metabolism; light pink—protein processing in endoplasmic reticulum; light yellow—glycerophospholipid metabolism; grey—unannotated). (**b**,**e**) The volcano plot of expressed genes in H- and L-type strains upon exposure to heat, respectively. (**c**,**d**) The top 10 enriched GO terms of up- and down-regulated genes in H-type strains upon exposure to heat, respectively. (**f**,**g**) The top 10 enriched GO terms of up- and down-regulated genes in H-type strains upon exposure to heat, respectively.

The sequencing data are summarized in Table S3. The pairwise correlation of all samples based on transcriptome data is showed in Figure S2. Transcripts with at least a 2-fold change in abundance and with a Benjamini–Hochberg-adjusted *p* value < 0.05 were regarded as differentially expressed genes (DEGs). A total of 2329 (1030 upregulated and 1299 downregulated) and 2834 (1206 upregulated and 1628 downregulated) DEGs were identified in H- and L-type strains upon exposure to thermostress, respectively (Table S4). The OPLS-DA model (CV-ANOVA, *p* < 0.05) showed clear gene expression patterns separating the H- and L-type *L. edodes* strains (Figure 3a). The volcano plots show that the top five down- and up-regulated genes in the H-type strain were different to those top five genes in the L-type strain, except for gene with identified as HHX47_DHR5000174, which was up-regulated in both strains under heat stress (Figure 3b,e). All the identified up- and down-regulated DEGs were annotated using KEGG enrichment analysis. The results showed that the up-regulated genes in the H-type strain under heat stress were mostly enriched (padjust < 0.05) in the intrinsic component of the membrane, the cellular anatomical entity, the integral component of the membrane, and the cellular component, while the down-regulated genes were enriched in catalytic activity, oxidoreductase activity, monooxygenase activity, and DNA repair (Figure 3c,d). For the L-type strain, heat stress induced the up-regulation of genes associated with the extracellular region, cellulose, polysaccharide and carbohydrate binding, integral and intrinsic components of the membrane, etc., and down-regulated genes involved in enzyme activities, proteasome regulatory particles, base subcomplexes, and FAD binding (Figure 3f,g). The KEGG analysis of the DEGs of H- and L-type strains upon exposure to heat is shown in Figure S3.

### 3.4. H- and L-type L. edodes Strains Showed Different Metabolic Profiles in Response to Heat Stress

**Figure 4:**
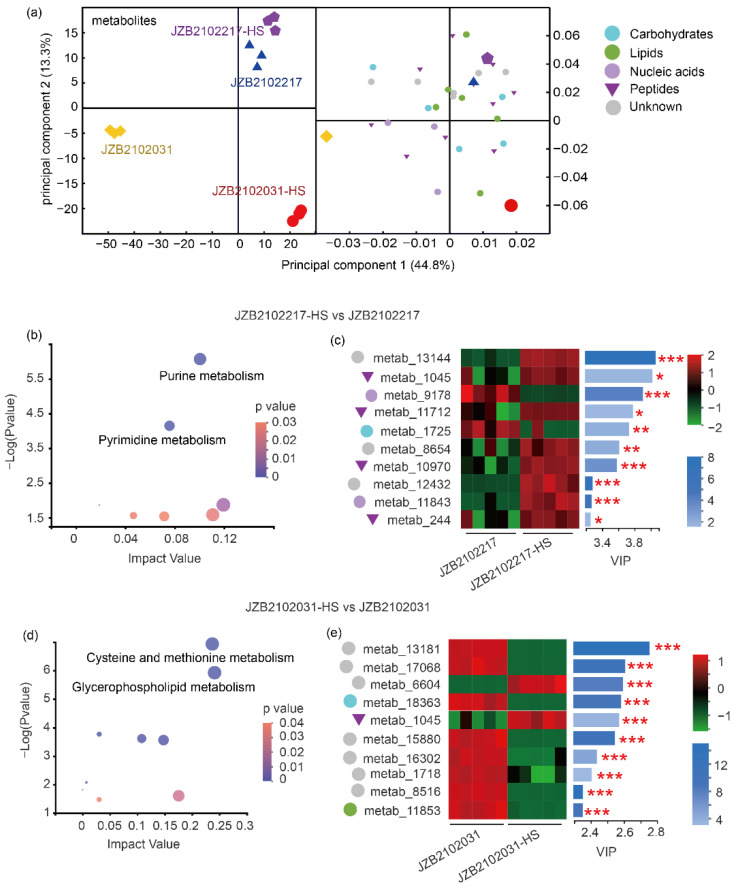
Metabolic profiling of H-type *L. edodes* strain JZB2102217 and L-type strain JZB2102031 in response to heat stress. (**a**) OPLS-DA analysis of the metabolome data of all samples (R2Xcum = 0.857, R2Ycum = 0.98, Q2cum = 0.899, CV-ANOVA, *p* = 2.083 × 10^−11^). Loading plot shows the top 30 metabolites (VIP > 1); (**b**,**c**) the KEGG topology analysis of DEMs and top 10 metabolites in H-type strain in response to heat stress (37 °C), respectively; (**d**,**e**) the KEGG topology analysis of DEMs and top 10 metabolites in L-type strain in response to heat stress (37 °C), respectively. The color of the blue bar indicates the value of -log10 (*p*-value); *, **, and *** indicate the abundance of compounds that were significant at *p* value < 0.05, <0.01, and <0.001, respectively.

The OPLS-DA model based on the metabolomes of the H- and L-type strains showed both clear strain-derived and heat stress-derived metabolic variations (Figure 4a). The Top 30 metabolites shown in the loading plot (VIP > 1) were classified as carbohydrates, lipids, nucleic acids, and peptides using KEGG analysis (Figure 4a). For the H-type strain, the heat-induced DEMs (FDR < 0.05) were largely enriched in purine and pyrimidine metabolism (Figure 4b). Of the top 10 heat-induced metabolites in the H-type strain, 4 were peptides and 8 were up-regulated (Figure 4b). For the L-type strain under heat stress, the DEMs were mainly enriched in cysteine and methionine metabolism and in glycerophospholipid metabolism (Figure 4d). Most of the top 10 heat-induced compounds that discriminated the L-type strain under heat stress were down-regulated and unannotated based on the KEGG database (Figure 4e).

Upon exposure to heat, 394 DEMs were identified in the L-type strain, while only 24 DEMs were identified in the H-type strain when the selection criteria for DEMs were narrowed to FDR < 0.05, VIP of OPLS-DA > 1, and FC > 1.2 (Figure S4a,b). A Venn network shows the most unique and common DEMs for the H- and L-type strains in response to thermostress (Figure S4c).

### 3.5. Integrative Analysis of Transcriptome and Metabolome of L. edodes under Heat Stress

**Figure 5:**
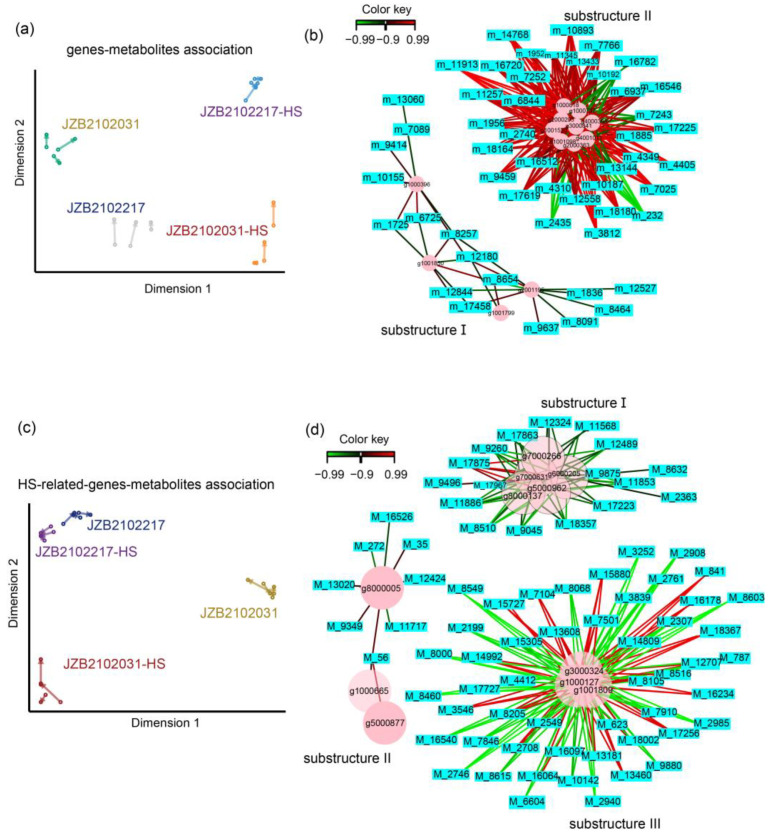
Transcriptome–metabolome-wide association analysis of all samples. (**a**) Sparse partial least squares (sPLS) association analysis between all genes and metabolites from all samples; (**b**) integration network of highly associated genes and metabolites at threshold of 0.9; (**c**) sPLS association analysis between thermotolerance-related genes and all metabolites from all samples; (**d**) integration network of highly associated thermotolerance-related genes and metabolites at threshold of 0.9. For the metabolite ID, “metab_” in Table S2 is abbreviated to “M_” for better readability.

Sparse partial least squares (sPLS) regression was performed to examine the relationships between the transcriptome and metabolome data of all the samples. The results showed that the transcripts were strongly associated with metabolites (Figure 5a). Further, interaction networks were constructed to infer the substructures of highly correlated genes and metabolites using a threshold of 0.9 (Figure 5b). The results showed that two clear interaction substructures could be detected without any intermediate elements. Interestingly, most of the correlations in substructure I, consisting of nine genes and 35 metabolites, were positive, while most of the correlations in substructure II, containing four genes and 16 metabolites, were negative (Figure 5b).

To better understand the regulatory network of transcripts and metabolites in response to heat stress, 18 protentional heat-tolerance (HT)-related genes were selected based on either GO ontology, or KEGG or pfam annotations. These HT-related genes were further subjected to sPLS analysis with all the detected metabolites. Strong associations were found between HT-related genes and metabolites (Figure 5c). From the correlation network, we identified three clear HT–gene–metabolite regulatory substructures (Figure 5d). In substructure I, the gene HHX47_DHR7000266 related to HSP binding, the gene HHX47_DHR7000631 related to glutathione peroxidase activity, the gene HHX47_DHR6000205 related to cell redox homeostasis, the gene HHX47_DHR5000962 encoding HSP 90, and the gene HHX47_DHR8000137 encoding HSP 70 were highly correlated with 17 metabolites; in substructure II, HHX47_DHR8000005 encoding HSP70 was associated with eight compounds, and another HSP70-encoding gene, HHX47_DHR000665, and an HSP20-encoding gene, HHX47_DHR5000877, were solely associated with the monosaccharide compound M_56 (N-Acetyl-D-quinovosamine); substructure III was the largest gene–metabolite association, consisting of three HS-related genes and 50 metabolites (Figure 5d).

## 4. Discussion

Generally, based on the temperature for fruiting, *L. edodes*, including both the wild and cultivated strains, could be divided into the groups H (high-temperature)-type, M (medium temperature)-type, L (low-temperature)-type, and B (broad-temperature)-type [11,12]. The temperature type of *L. edodes* is usually obtained by observing the fruiting temperature during cultivations under natural climatic conditions [11,49]. The temperature type of *L. edodes* could, moreover, be characterized by other strain-typing approaches such as inter-simple sequence repeat (ISSR) analysis [12] and amplified fragment length polymorphism (AFLP) analysis [10]. These phenotypic divergence- and molecular marker-based results suggest strong genetic divergences underlying the temperature type of *L. edodes*.

Except for characterization from the aspect of the phenotype, the genetic basis underlying the temperature type of *L. edodes* is largely unknown. Here, using comparative transcriptomics and metabolomics, we show, for the first time, that the H- and L-type *L. edodes* strains possess distinct transcriptional and metabolic patterns under optimum temperature conditions, which underlie the genetic divergence of the temperature type. Similarly, a recent population genomics study shows that temperature is the key environmental factor involved in the genetic divergence and phenotypic differentiation of *L. edodes* [14]. Our data show that the H-type strain had a higher expression level in the genes involved in toxin processes and cellulose, polysaccharide, and carbohydrate binding, while it had a lower level in the genes involved in oxidoreductase activity, protein-disulfide reductase activity, etc. Oxidoreductase activity has been found to be involved in light-induced brown-film formation in *L. edodes* [50,51,52]. From the aspects of metabolism, our data show that the H-type *L. edodes* strains accumulated more metabolites in the pathways of starch and sucrose metabolism and arginine and proline metabolism, while the L-type strain accumulated more metabolites in glycerophospholipid and purine metabolism. Through we have found some interesting differences in gene expression and metabolism between different temperature types of the *L. edodes* strain, we are still a long way from building an explicit connection between genes and temperature type. Nevertheless, the current paper represents pioneering work that deepens our understanding of the genetic basis and metabolic processes regarding temperature type.

The optimum growth temperature for *L. edodes* mycelium ranges from 24 to 27 °C and the lethal temperature could be 38 °C or above, depending on the strain [26]. Heat stress appears to be the major abiotic constraint inhibiting mycelium growth, disease resistance, and fruiting body development, thereby seriously reducing fruiting body productivity and the quality of *L. edodes* [24,53,54,55]. Therefore, it is of great significance to decipher the mechanisms by which *L. edodes* addresses thermostress. For *L. edodes*, the optimum temperature required for fruiting body induction is different from that for vegetative growth. However, whether the temperature type is associated with the optimum mycelium growth temperature for *L. edodes* remains unknown. In this study, we compared the physiological and phenotypic responses of H- and L-type *L. edodes* strains under exposure to heat stress. We found that the growth rate of the L-type strain decreased more and had a higher growth inhibition rate after recovery than that of the H-type strain in response to heat stress, suggesting that the L-type strain might be more sensitive to heat stress. Though the temperature-type is defined by the fruiting temperature rather than the temperature at which mycelia grow [12], we found distinct responses of the H- and L-type *L. edodes* strains in their gene expression and metabolism under heat stress. Moreover, Wang et al. reported that the high-temperature type *L. edodes* strain could form a fruiting body at a higher temperature and had a higher fruiting body yield than the low-temperature type of *L. edodes* [56,57]. Taken together, it is interesting that the temperature types of *L. edodes* could be associated with the thermotolerance of mycelium. Thus, we suggest a large-scale evaluation on the thermotolerance of *L. edodes* strains with different temperature types.

A considerable number of studies have accumulated over the years addressing the topic of heat resistance in the mycelium of *L. edodes* [15,26,55]. Previous studies have documented that heat shock proteins (HSPs), indoleacetic acid (IAA), catalase, and trehalose play crucial roles in the thermotolerance of *L. edodes* [15,29,30]. HSPs are a family of conserved proteins that are up-regulated at high temperatures and play a crucial role in protein renaturation, enzyme and membrane stability, and cell homeostasis [58,59]. In this paper, we found that 13 *Hsp20* genes were significantly up-regulated in response to heat stress in both the H- and L-type *L. edodes* strains, highlighting the importance of HSPs for *L. edodes* to cope with heat stress. More recently, it was reported that overexpression of the *Agaricus bisporus Hsp20* gene enhanced the mycelial thermotolerance of *L. edodes* [55]. Moreover, the HSP40 protein LeDnaJ07 was also proven to be integral to conferring heat resistance on *L. edodes* [15,29,30].

The central outcome of our study is that the H- and L-type *L. edodes* strains respond differently upon exposure to heat stress. To guarantee a more representative assessment, we selected two typical H- and L-type strains which are widely cultivated in northern and southern China, respectively. However, we still suggest more comprehensive investigations to dig deeper in their temperature-type-dependent thermotolerance mechanisms.

Though the explicit mechanisms by which *L. edodes* copes with heat remain to be elucidated, our data suggest, for the first time, that the molecular strategy for addressing heat can be temperature-type-dependent and provide informative clues for further relevant studies.

## Data Availability

The raw RNA-seq data and LC-MS data of the *L. edodes* strains are available at the National Genomics Data Center, China National Center for Bioinformation, under the BioProject IDs PRJCA014125 and PRJCA014136, respectively.

## Supplementary Materials

The following supporting information can be downloaded at: https://www.mdpi.com/article/10.3390/jof9020179/s1, Figure S1: KEGG enrichment analysis of DEGs between H-type strain JZB2102217 and L-type strain JZB2102031; Figure S2: Correlation of different samples based one RNA-seq data; Figure S3: KEGG enrichment of DEGs of H-type strain JZB2102217 and L-type strain JZB2102031 in response to thermostress; Figure S4: Volcano plot, Venn network, and KEGG enrichment for DEMS of H-type strain JZB2102217 and L-type strain JZB2102031 in response to thermostress; Table S1: Expression level and annotation of all expressed genes (RPKM > 10); Table S2: Abundance and annotations of all detected metabolites; Table S3: Overview of the RNA-seq data; Table S4: Number of DEGs in H-type strain JZB2102217 and L-type strain JZB2102031 in response to thermostress.
