# Supplementary material for: Two Strains of Lentinula edodes Differ in Their Transcriptional and Metabolic Patterns and Respond Differently to Thermostress
Source: J Fungi (Basel). 2023 Jan 29;9(2):179. doi: 10.3390/jof9020179 (PMC9961724; doi:10.3390/jof9020179)
Supplement: Supplementary file 1 [file jof-09-00179-s001.zip › Supplementary Materials.docx]

Figure S1. KEGG enrichment analysis of un-regulated (a) and down-regulated (b) genes between JZB2102217 and JZB2102031.

Figure S2. Correlations of different samples based on rna-seq data of all samples.

Figure S3. KEGG enrichment of up- and down-regulated gene of JZB2102217-HS vs JZB2102217 (a, b) and JZB2102031-HS vs JZB2102031 (c, d).

Figure S4. Volcano plot (a, b), venn network (c) and KEGG enrichments (d, e) for DEMS ofJZB2102217-HS vs JZB2102217 and JZB2102031-HS vs JZB2102031.

Table S1. Expression level and annotation of all expressed genes (RPKM $\mathbf{>}$10 in at least on samples).

Table S2. Abundance and annotations of all detected metabolites.

Table S3. Overview of the statistics of the rna-seq data.

Table S4. Number of DEGs of JZB2102217-HS vs JZB2102217 and JZB2102031-HS vs JZB2102031.
