# Supplementary figures and images for: Two Strains of Lentinula edodes Differ in Their Transcriptional and Metabolic Patterns and Respond Differently to Thermostress
Source: J Fungi (Basel). 2023 Jan 29;9(2):179. doi: 10.3390/jof9020179 (PMC9961724; doi:10.3390/jof9020179)

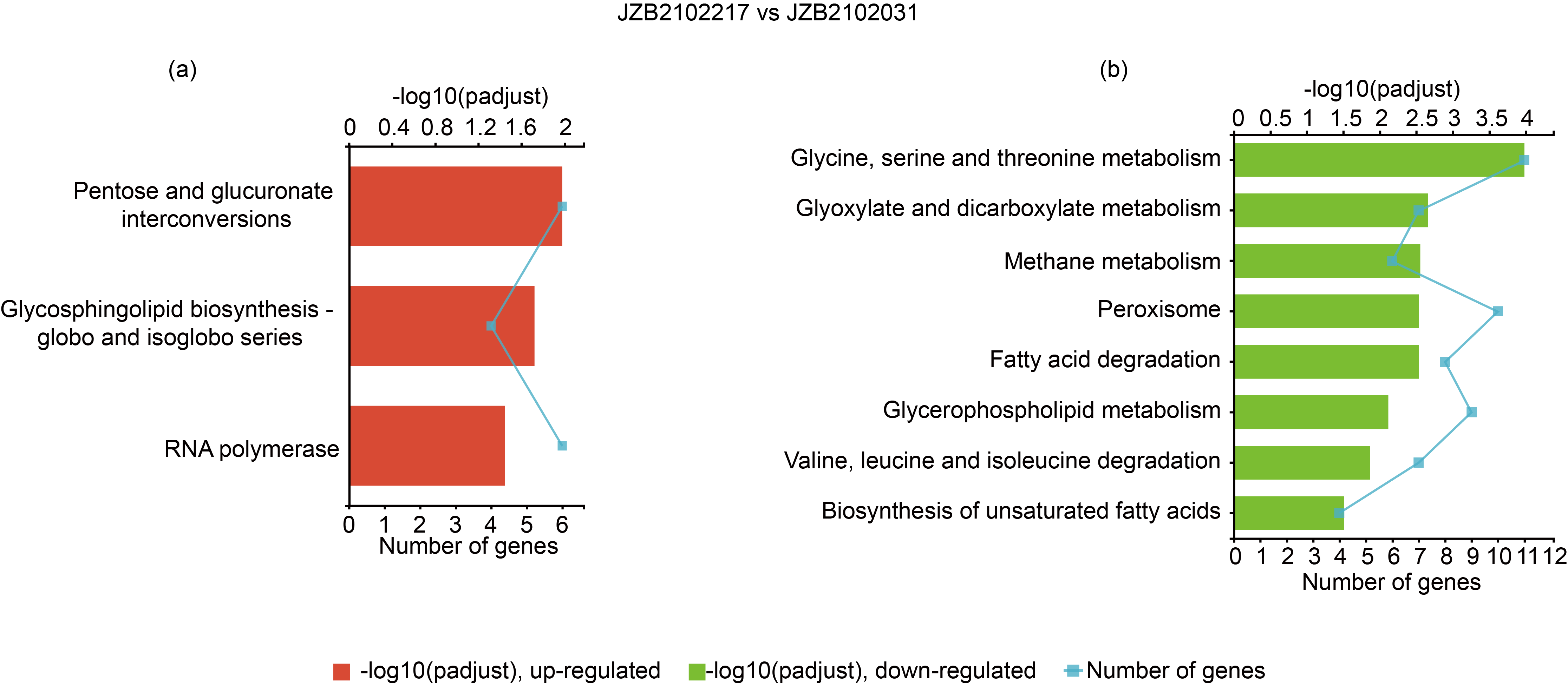

Supplement: Supplementary file 1 [file jof-09-00179-s001.zip › Figure S1_KEGG enrichemnt analysis of DEGs between JZB2102217 and JZB2102031.tif]

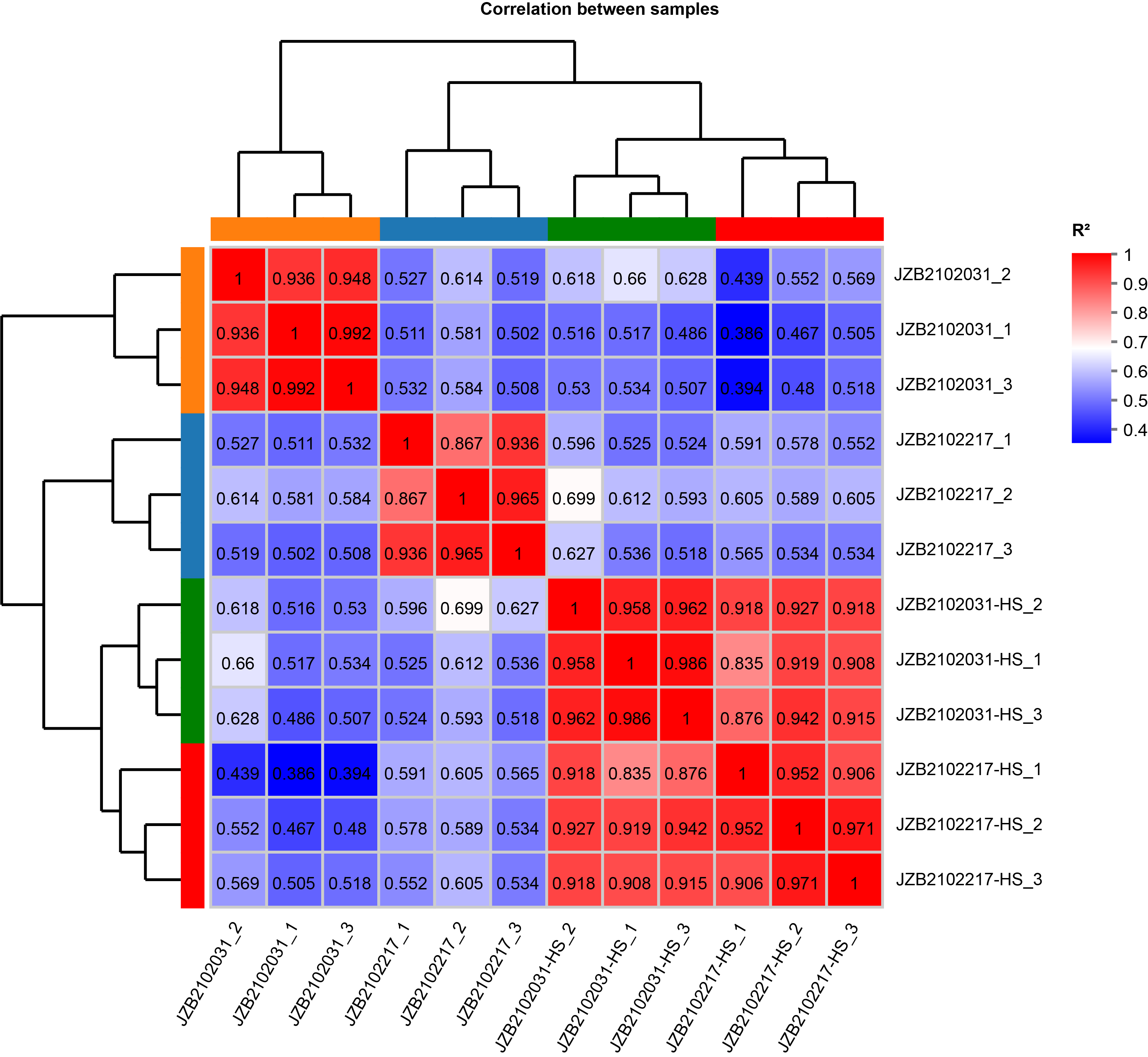

Supplement: Supplementary file 1 [file jof-09-00179-s001.zip › Figure S2 correlation of different samples based one rna-seq data.tif]

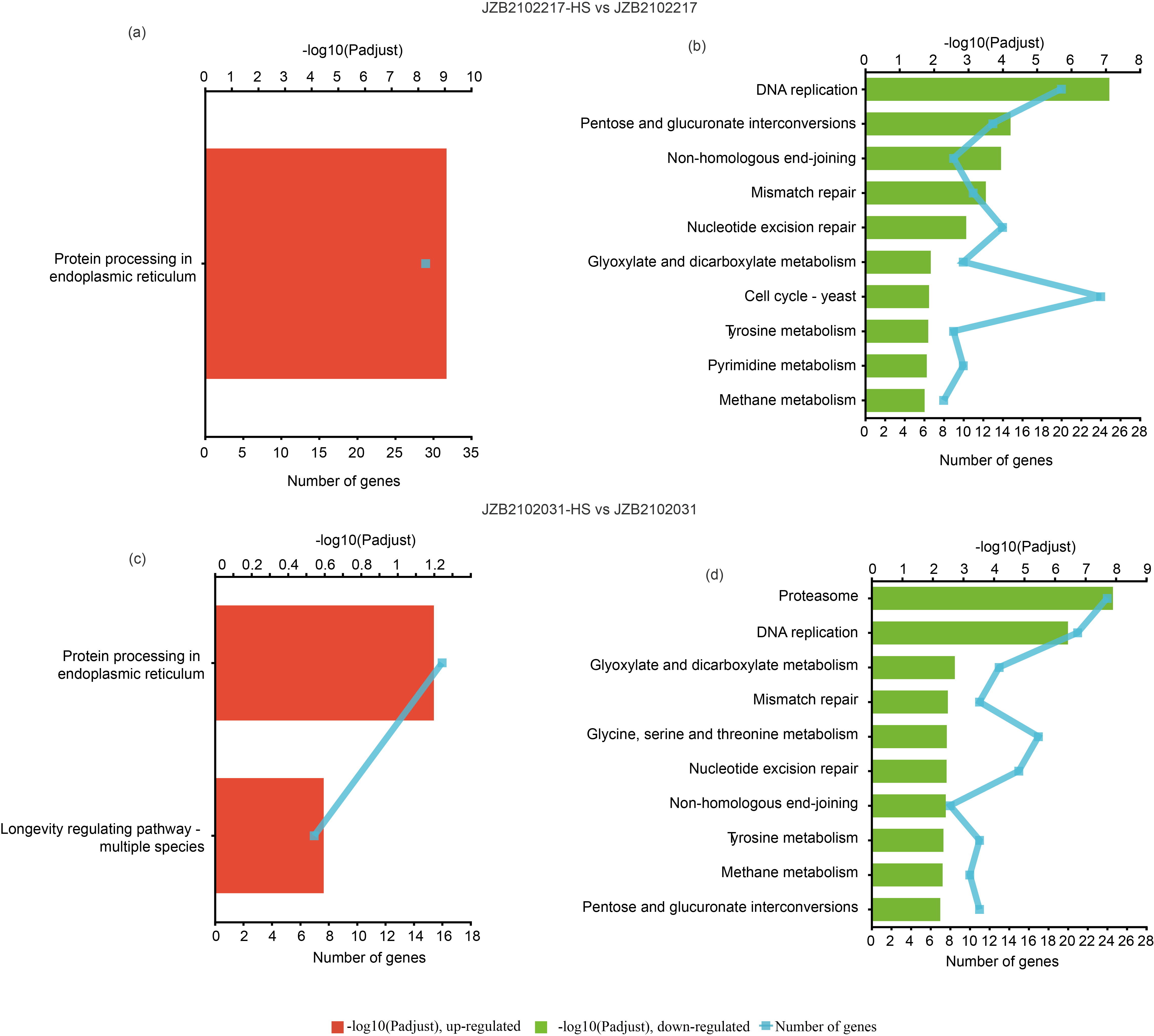

Supplement: Supplementary file 1 [file jof-09-00179-s001.zip › Figure S3 KEGG enrichment of DEGs of JZB2102217-HS vs JZB2102217 and JZB2102031-HS vs JZB2102031.tif]

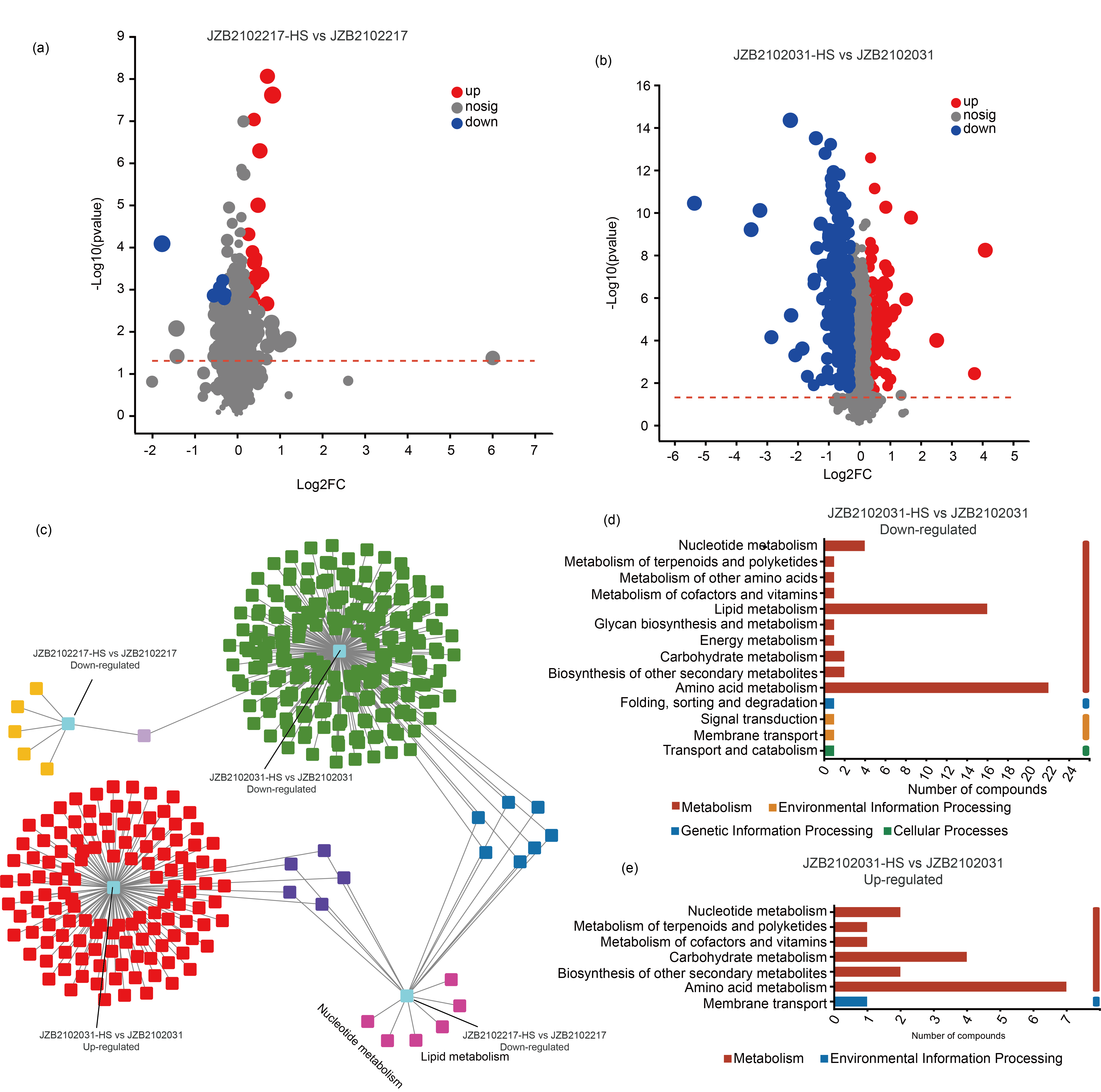

Supplement: Supplementary file 1 [file jof-09-00179-s001.zip › Figure S4 volcano plot, venn network and KEGG enrichemnt for DEMS ofJZB2102217-HS vs JZB2102217 and JZB2102031-HS vs JZB2102031.tif]
